# Surfactin: A Quorum-Sensing Signal Molecule to Relieve CCR in *Bacillus amyloliquefaciens*

**DOI:** 10.3389/fmicb.2020.00631

**Published:** 2020-04-30

**Authors:** Bing Chen, Jiahong Wen, Xiuyun Zhao, Jia Ding, Gaofu Qi

**Affiliations:** College of Life Science and Technology, Huazhong Agricultural University, Wuhan, China

**Keywords:** *Bacillus amyloliquefaciens*, surfactin, quorum-sensing, carbon catabolite repression, catabolite control protein A

## Abstract

*Bacillus* utilize preferred sugars such as glucose over other carbon sources due to carbon catabolite repression (CCR). Surfactin is a small signal molecule to regulate the quorum-sensing (QS) response such as biofilm formation and sporulation in *B. subtilis*. Here, the *srfA* operon for synthesis of surfactin was mutated for disrupting the production of surfactin in *B*. *amyloliquefaciens.* The *srfA*-mutant strain showed a defective biofilm and sporulation but could be restored by addition with surfactin, indicating that surfactin is a QS signal molecule in *B*. *amyloliquefaciens*. Unexpectedly, mutation of *srfA* also led to the cells’ death although nutrients were still enough to support the bacterial growth during this period. Analysis of transcriptomes found that the *srfA*-mutant strain could not relieve CCR to use non-preferred carbon sources after glucose exhaustion due to deficiency of surfactin. This was further verified by the fact that addition with glucose could dramatically restore the growth, and addition with surfactin could improve the enzymes’ activity (e.g., glucanase and α-amylase) to use non-preferred carbon sources in the *srfA-*mutant strain. After glucose exhaustion, the cells produce surfactin to relieve CCR for utilizing non-preferred sugars. As a signal molecule to regulate QS, surfactin also directly or indirectly relieves the CcpA-mediated CCR to utilize non-preferred carbon sources countering nutrient limitation (e.g., glucose deprivation) in the environment. In conclusion, our findings provide the first evidence that the QS signal molecule of surfactin is also involved in relieving the CcpA-mediated CCR in *B. amyloliquefaciens*.

## Introduction

*Bacillus* are generally soil-dwelling Gram-positive bacteria ubiquitously distributed in the natural environment. Many *Bacillus* species, such as *B. subtilis*, *B. licheniformis*, and *B. amyloliquefaciens*, are used as hosts for making fermentation products and microbial pesticides. *B. subtilis* is also used as a model system for Gram-positive bacteria. To survive in the complex environment, *Bacillus* metabolize glucose as a primary carbon source. Upon deprivation, many genes and proteins will specifically respond to glucose starvation, including the genes required for transport and utilization of alternative carbon sources, as well as the genes that allow employment of survival strategies like quorum sensing (QS) (de Jong et al., 2012). However, how to link utilization of alternative carbon sources and QS is still unknown in *Bacillus*.

*Bacillus* utilize a large number of carbon sources, but a well-known phenomenon is that the most favored sugars (e.g., glucose, fructose, or malate) are used preferentially over other carbon sources. The mechanism of preferentially utilizing carbohydrates is complex, containing multiple specific and global regulatory strategies strictly controlled by carbon catabolite repression (CCR). By CCR, *Bacillus* first utilize preferred carbohydrates in the presence of non-preferred sources with significant quantities that is beneficial for a successful competition with other microorganisms in the natural environment (Marciniak et al., 2012; Kim and Burne, 2017). In *B. subtilis*, CCR is mainly exerted by a global transcription factor of CcpA (catabolite control protein A) (Yang et al., 2017). In the presence of preferable carbon sources such as glucose, the histidine-containing phosphotransfer protein (HPr) is phosphorylated by HPr kinase (HPrK) at Ser_46_ by the bifunctional HPr kinase/phosphorylase HPrK/P at high fructose-1,6-bisphosphate and ATP or pyrophosphate concentrations and then forms a complex with CcpA that can bind to a conserved *cis*-acting sequence called catabolite responsive elements (*cre*) to repress the gene transcription involved in catabolism of non-preferred carbon sources (Schumacher et al., 2011; Ishii et al., 2013; Charbonnier et al., 2017; Kim and Burne, 2017). After glucose exhaustion, HPrK/P dephosphorylates HPrSer_46_P in the presence of high inorganic phosphate (Pi) concentrations by phospho-phosphorylation of the Pi. The CcpA–HPrSer_46_P complex has a weak binding constant KA. Therefore, free HPrSer_46_P is present in the cell in excess to CcpA to keep CcpA saturated with HPrSer_46_P. In the presence of a high Pi concentration, HPrK/P dephosphorylates free HPrSer_46_P until the HPrSer_46_P concentration is so low that HPrSer_46_P dissociates from CcpA, and in consequence, CcpA dissociates from *cre* (Mijakovic et al., 2002; Poncet et al., 2004; Fujita, 2009).

With nutrient limitation (e.g., glucose deprivation), *Bacillus* will initiate a survival strategy called QS, by which the cells cooperate in a density-dependent manner through secretion and detection of specific autoinducer molecules such as ComX (Even-Tov et al., 2016; Pollak et al., 2016). In *B. subtilis*, ComX is a component of QS system encoded by the *comQXPA* operon (Tran et al., 2000; Stefanic and Mandic-Mulec, 2009), which contains an isoprenyl transferase of ComQ, a signal peptide of ComX, and the two-component system of ComP (histidine kinase) and ComA (response regulator) (Ansaldi et al., 2002; Ansaldi and Dubnau, 2004; Oslizlo et al., 2014). Pre-ComX is initially synthesized and then processed and modified by ComQ. Upon reaching a threshold concentration, ComX binds following leads to ComP autophosphorylation. ComP∼P transfers the phosphoryl group to ComA (Schneider et al., 2002), which controls the expression of *srfA* encoding a very large protein complex for non-ribosomal synthesis of the lipopeptide surfactin (Hamoen et al., 2003; Stefanic and Mandic-Mulec, 2009; Oslizlo et al., 2014). Surfactin is one of the most effective biosurfactants discovered so far (Stefanic and Mandic-Mulec, 2009; Oslizlo et al., 2014), as well as a signal molecule to trigger QS in *B. subtilis* (López et al., 2009a). Surfactin can cause pores on cell membrane leading the leakage of K^+^ from cytoplasm that acts as a signal to activate the membrane histidine kinase KinC and eventually phosphorylate Spo0A, a global regulator of QS in *B. subtilis* (López et al., 2009b; Banse et al., 2011; Bendori et al., 2015). High levels of Spo0A∼P are required for sporulation while low levels of Spo0A∼P are essential for biofilm formation (López et al., 2009b, 2010; Vlamakis et al., 2013).

In the natural environment, available carbon sources are a mixture of diverse carbohydrates including both of hexose and pentose sugars like glucose, xylose, arabinose, etc. (Li et al., 2014). CCR enables competitive advantages in carbon catabolism. By CCR, *Bacillus* utilize, out of a mixture of compounds, preferred carbon source (e.g., glucose) to allow a fast growth (Fujita, 2009). Accordingly, glucose content might be a restrictive nutrient factor for cell density in a given environment. Consequently, we hypothesized that after glucose exhaustion, CCR will be relieved to utilize non-preferred sugars supporting the initiated QS response such as biofilm formation and sporulation in *Bacillus*.

In addition to surfactin, *Bacillus* also produce other lipopeptides such as iturin and fengycin, which can interact with the biological membrane of pathogenic fungi to induce cell leakage and death (Koumoutsi et al., 2004; Zeriouh et al., 2014). Thereby, *Bacillus* are popularly used as biological control agents by virtue of their lipopeptide products (Xu et al., 2013; Zeriouh et al., 2014; Yang et al., 2015). Previously, we isolated a strain of *B. amyloliquefaciens* WH1 with production of several lipopeptides such as surfactin, iturin, and fengycin (Qi et al., 2010). In this study, we mutated the gene cluster for biosynthesis of surfactin (*srfA*), fengycin (*fenA*), and iturin (*ituB*), respectively. Unexpectedly, mutation of *srfA* caused a seriously defective growth in WH1. Further analysis found that surfactin, a signal peptide to regulate QS, is also involved in relieving CCR in *B. amyloliquefaciens*, by which QS is tightly linked with CCR to counter carbon sources limitation in the natural environment.

## Materials and Methods

### Bacterial Strains, Plasmids, Primers, and Reagents

Experiments were performed with the strains and plasmids in Supplementary Table S1. Oligonucleotide primers, listed in Supplementary Table S2, were designed on the genome of *B. amyloliquefaciens*. Materials for DNA manipulation including T4 DNA ligase, DNA marker, *Pfu* DNA Polymerase, and Plasmid Miniprep Kit were from Takara Bio (China). Nucleotide sequences were determined by Beijing Genomics institution (China). Surfactin was chemically synthesized by Chinese Peptide Company (China) (Zhang et al., 2017). Bacitracin was from Lifecome Biochemistry Co., Ltd., China. Amphotericin B and Nystatin were purchased from Biosharp, China. All other chemicals were of analytical grade supplied by Sinopharm Chemical Reagent (China).

### Construction of Mutant Strains

Genes including *srfA, ituB*, *fenA*, *comA*, and *ccpA* were mutated by double crossover homologous recombination method (Qi et al., 2014). Firstly, two approximately 500-bp arms homologous to the 5′ and 3′ coding region of the above genes were amplified by PCR from *B. amyloliquefaciens* WH1 by primers LF and LR for L arm and RF and RR for R arm (Supplementary Table S2) and then ligated by splicing with Overlapping Extension PCR (SOE-PCR) with primers LF and RR, respectively. After digestion by *Bam*HI and *Xba*I, the DNA fragments were subcloned into the vector T2(2) with a temperature-sensitive replicon from *B. subtilis* (Qi et al., 2014).

The resulting plasmids were transformed into *B. amyloliquefaciens* WH1 by our previous methods (Qi et al., 2014). Transformants were selected by kanamycin resistance (20 μg/ml) and then verified by PCR using LF and RR primers (Supplementary Table S2). The selected transformants were cultured in LB medium containing kanamycin at 45°C for 8 h to promote the first crossover and then the mutants with first crossover were selected by PCR with single-crossover LF and RR primers (Supplementary Table S2). Thereafter, the selected mutants with single crossover were cultured in LB medium at 37°C for 8 h and then the cultures were serially diluted following spread on LB agar plates for 100 μl/plate. After being cultured at 37°C for 24 h, the colonies were picked up and replicated on kanamycin plates for selecting sensitive ones. Knockout strains were screened out and had looped out of the kanamycin-resistant gene by the second crossover and confirmed by PCR with LF and RR primers (Supplementary Table S2) followed by nucleotide sequencing of the PCR products.

### Analysis of Phenotype

The wild-type and mutant strains were cultured on LB agar plates and then the morphology of colonies was observed by microscope. Robust pellicle (floating biofilm) was determined in 24-well plates with 2 ml of LB medium each well. The multiwell plates were inoculated with different strains and then cultured at 28°C for 48 h to allow float biofilm formation.

The growth of different strains was detected in LB medium. The strains were incubated at 28°C and 180 rpm and then the OD_600_ value of culture was determined by a spectrophotometer, and the cells collected at several time points (18, 20, 26, and 29 h) were stained by crystal violet following observation by microscope. WH1 and Δ*srfA* (*srfA*-mutant strain) were also respectively incubated at 28°C and 180 rpm with a ventilation of 3 L/min in a 5-L bioreactor (National center of Bio-Engineering and Technology, China) and then the dissolved O_2_ of culture was recorded by a sensor.

### Analysis of Lipopeptides

Lipopeptides were crudely purified from the culture of different strains. Briefly, the pH of culture supernatant was adjusted to 4.0 for precipitation of proteins and then to 2.0 for precipitation of lipopeptides. After centrifugation, the pellets were collected and dissolved in water and then extracted by the same volume of *n*-butanol for determination of the lipopeptides by MALDI-TOF (Qi et al., 2010). Additionally, the agar plates containing sheep blood cells were used for culturing the wild-type and mutant strains and then the hemolytic activity was determined to evaluate the surfactin production in different strains (Shaligram and Singhal, 2010).

### Effect of Surfactin and Antibiotics on Bacterial Growth and Biofilm Formation

Δ*srfA* was cultured in LB medium supplemented with surfactin or other antibiotics including bacitracin, amphotericin B, and nystatin (80 μg/ml) at 28°C and 180 rpm for 24 h and then the bacterial growth was recorded. Δ*srfA* was also cultured in 24-well plates with 2 ml of LB medium added with surfactin, bacitracin, amphotericin B, or nystatin at different concentrations (0, 20, 40, 60, and 80 μg/ml) in each well at 28°C for 48 h to allow biofilm formation. The *comA*-null mutant strain (Δ*comA*) was also cultured in LB medium supplemented with surfactin (80 μg/ml) as above and then the bacterial growth was observed.

### Comparison of Transcriptomes Between WH1 and ΔsrfA

Transcriptome analysis was performed by SHBIO Technology Co., Ltd. (China). WH1 and Δ*srfA* were cultured in LB medium at 28°C and 180 rpm for 18 h (the highest biomass of Δ*srfA* at this time point). Culture (1.5 ml) of three test tubes per replicate was centrifuged for collecting cell pellets and then used for isolating RNA by RNeasy Mini Kit (Qiagen, Germany). Enrichment of mRNA was done with the RiboZero rRNA Removal Kit for Bacteria according to the manufacturer’s directions. Library preparations for paired-end sequencing on the Illumina HiSeq 1500 platform were performed according to the protocols of Illumina, and the raw Illumina pair-end read data for all samples were deposited in the Short Read Archive under the Bioproject: PRJNA560470.

Obtained sequences were mapped onto the reference genome of *B. amyloliquefaciens* (NCBI Accession no: CP000560.1). To quantify gene transcription from the obtained sequence reads, RPKM values (Reads Per Kilobase per Million mapped reads) were calculated using the number of reads mapped to a gene, the total amount of mapped reads in the experiment, and the length in base pairs for a gene, and then used for comparison of normalized gene transcription rates. Differential gene transcriptions between Δ*srfA* and WH1 were evaluated using the software platform ReadXplorer. The thresholds for significant changes in gene transcription were fold-changes of greater than 2 or less than 0.5 with an adjusted *p*-value of 0.05 (Kröber et al., 2016). By KEGG analysis, the metabolic pathways were constructed to describe the differential gene expression (DGE) involved in carbon metabolism between these two strains.

Some key gene transcriptions involved in carbon metabolism were quantified by quantitative real-time PCR (qRT-PCR). RNA was extracted as above and then cDNA was produced by reverse transcription with 1 μg RNA, iScript Select cDNA Synthesis Kit, and random oligonucleotide primers (Bio-Rad). qRT-PCR was performed with cDNA, SsoAdvanced Universal SYBR Green Supermix (Bio-Rad), and target-specific primers (Supplementary Table S3) in CF96 Real-Time System (Bio-Rad) as follows: 1 cycle of 95°C for 10 min, followed by 40 cycles of 95°C for 15 s, and 46°C for 20 s. All expression data were normalized to the copy number of 16S rRNA in each sample.

### Effect of Glucose on Bacterial Growth

The glucose content in LB medium was enzymatically detected using SBA-40D Bio-analyzer (Shandong Academy of Sciences, China). Δ*srfA* was cultured at 28°C and 180 rpm in LB medium, or LB medium supplemented with glucose (4 g/L), and then the bacterial growth was observed after 24 h. Δ*srfA* and WH1 were also cultured in M9 medium (5.92 mg MgSO_4_⋅7H_2_O, 0.11 mg CaCl_2_, 17.0 g Na_2_HPO_4_⋅12H_2_O, 3.0 g KH_2_PO_4_, 0.5 g NaCl, and 1.0 g NH_4_Cl in 1 L) with mixed sugars (0.10 g/L glucose and 3.90 g/L xylose) at 28°C and 180 rpm and then the bacterial growth was observed after 24 h.

### Detecting Activity of Amylase and Glucanase

The activity of α-amylase and glucanase in WH1 and Δ*srfA* was respectively determined by measuring the amount of reducing sugars released from soluble starch or glucan using alkaline DNS oxidant reagent (Kanchi et al., 2018). WH1 and Δ*srfA* were cultured in LB or LB added with surfactin (80 μg/ml) at 28°C for 15 h, and then the culture was centrifuged at 12,000 rpm and 4°C for collecting the supernatant as crude enzyme solution. Crude enzyme solution (0.5 ml) was mixed with 1 ml of 2% (w/v) soluble starch or glucan (20,000 Da), incubated at 37°C for 5 min, and then the reaction was stopped by addition of 1 ml of DNS reagent. The solution was incubated in boiling water bath for 5 min to form color and then the OD_540_ value was detected by a spectrophotometer after dilution with water. Measurements were also performed on a blank without substrate but with crude enzyme solution, and a control without enzyme solution but with substrate at the same time. Each measurement procedure was performed three times, with measurements taken in triplicate. The enzyme activity was defined as the amount of reducing sugar produced by enzymes in 1 ml of crude enzyme solution per minute (μg/ml.min).

The α-amylase activity was also determined on LB agar plates added with starch. Briefly, the wild-type WH1 and mutants of Δ*srfA*, Δ*ccpA* (*ccpA-*null mutant strain), and Δ*srfA*Δ*ccpA* (*srfA* and *ccpA* mutant strain) were cultured on LB agar plates containing 4 g/L of starch at 28°C for 24 h, and then the plates were stained by iodine to determine the bacterial α-amylase activity. The digested starch could not be stained by iodine showing a clear zone around bacterial colony on the plates.

### Statistical Analysis

All experiments are repeated in triplicate. Data between two groups were compared by a Student *t* test with a significance level of ^∗^*p* < 0.05 and ^∗∗^*p* < 0.01.

## Results

### Lipopeptide Production in Different Strains

The genes for biosynthesis of surfactin (*srfA*), iturin (*ituB*), and fengycin (*fenA*) were mutated in *B. amyloliquefaciens* WH1, respectively (Supplementary Figure S1). We determined the lipopeptide production in these strains by MALDI-TOF (Deng, 2009). The results showed that WH1 produced surfactin (1044), iturin (1081, 1095), and fengycin (1464, 1502); Δ*srfA* produced iturin (1043, 1081, 1095) and fengycin (1435, 1449, 1463, 1501); Δ*ituB* produced surfactin (1044) and fengycin (1435, 1464, 1502); and Δ*fenA* produced surfactin (1044) and iturin (1081) (Supplementary Figure S2A). Thereby, the mutant strains of Δ*srfA*, Δ*ituB*, and Δ*fenA* lose the ability to produce surfactin, iturin, and fengycin, respectively. However, it is surfactin rather than iturin and fengycin, with a strong hemolytic activity (Shaligram and Singhal, 2010). Here, WH1 showed an obvious hemolytic activity around colony as well as Δ*ituB* and Δ*fenA*, but the hemolytic activity disappeared in Δ*srfA* (Supplementary Figure S2B). The result further indicated that Δ*srfA* loses the ability to produce surfactin.

### Mutation of srfA Led to a Defective Biofilm Formation and Growth in *B. amyloliquefaciens*

**FIGURE 1:**
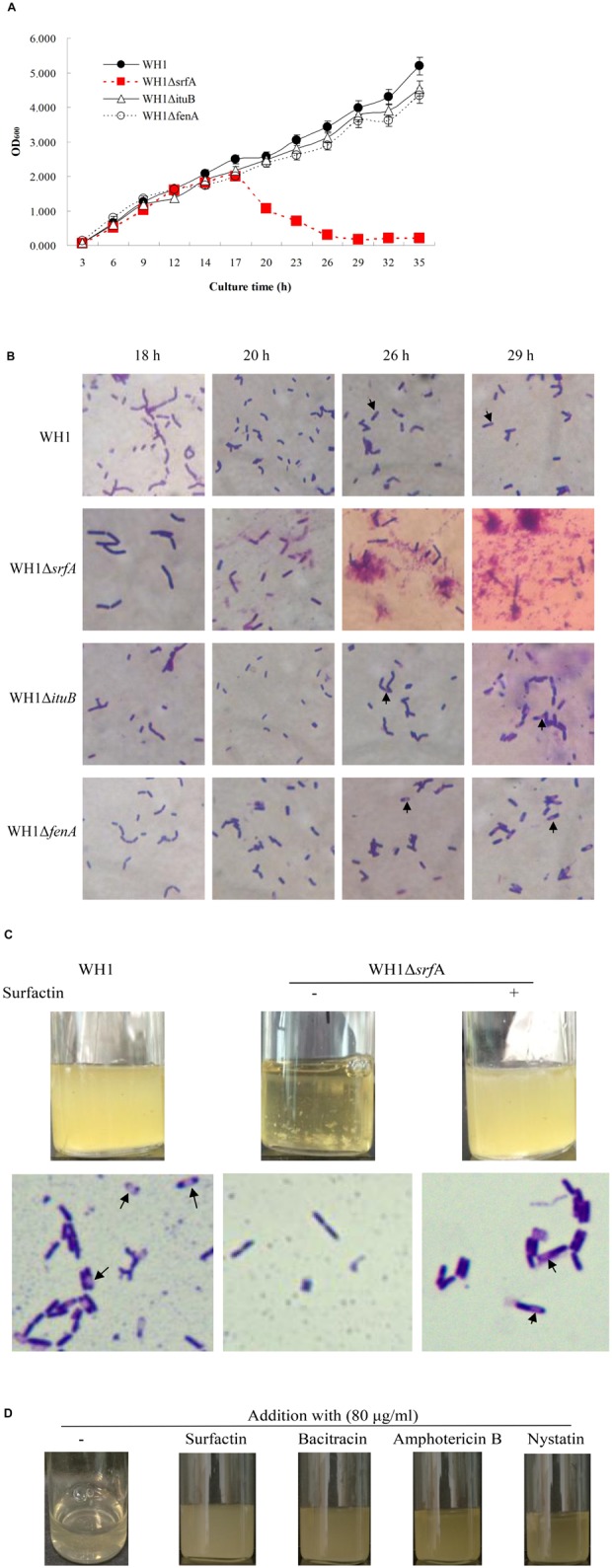
Mutation of *srfA* resulted in a defective bacterial growth. **(A)** Growth curves of WH1 and lipopeptide gene mutant strains. Mutation of *srfA* led to a defective bacterial growth. **(B)** Cells of WH1 and lipopeptide gene mutant strains (magnification 1000×). Δ*srfA* died after 18 h without sporulation while other strains grew well with sporulation. Arrows indicate the spores. **(C)** Addition with surfactin dramatically restored the growth and sporulation in Δ*srfA* (magnification 1000×). Spores are indicated by arrows. **(D)** Addition with bacitracin, nystatin, or amphotericin B partially restored the growth in Δ*srfA*.

The colony morphology of Δ*srfA* was flat and unwrinkled, very different from WH1, Δ*ituB*, and Δ*fenA* (Supplementary Figure S2C). We further investigated the biofilm formation of mutant strains in LB medium, and found that both Δ*ituB* and Δ*fenA* could form a robust pellicle like WH1, but Δ*srfA* only produced a thin and fragile pellicle (Supplementary Figure S2D). The result suggested that surfactin is required for the biofilm formation in *B. amyloliquefaciens*. Unexpectedly, mutation of *srfA* also resulted in a significantly different growth profile from WH1, Δ*ituB*, and Δ*fenA*. In 18 h, Δ*srfA* showed a similar growth profile to WH1. Thereafter, it fell fast into death while WH1, Δ*ituB*, and Δ*fenA* all grew well in this period (Figure 1A). WH1 could form spores as well as Δ*ituB* and Δ*fenA*, but Δ*srfA* could not sporulate in LB medium (Figure 1B).

The defective growth of Δ*srfA* could be restored by addition with surfactin in LB medium (Figure 1C). Addition with surfactin could also restore the sporulation (Figure 1C) and biofilm formation (Supplementary Figure S3) in Δ*srfA*. Besides, for surfactin, other antibiotics such as bacitracin, amphotericin, and nystatin could also effectively alleviate the death of Δ*srfA* due to lack of surfactin (Figure 1D). Except for Amphotericin B, both Bacitracin and Nystatin could also partially restore the biofilm formation in Δ*srfA*, but were weaker than surfactin (Supplementary Figure S3).

### Knockout of comA Resulted in a Similar Phenotype to ΔsrfA

ComA∼P is required for the transcription of *srfA* operon in *B. subtilis* (Gonźalez-Pastor, 2011). Here, we deleted *comA* in *B. amyloliquefaciens*. As expected, knockout of *comA* resulted in a similarly defective growth, biofilm formation, and colony morphology to Δ*srfA* (Supplementary Figure S4). Nevertheless, the defective growth of Δ*comA* could also be restored by addition with surfactin, which further verified that surfactin is very important for the growth of *B. amyloliquefaciens*.

### Disordered Carbon Metabolism in ΔsrfA

**FIGURE 2:**
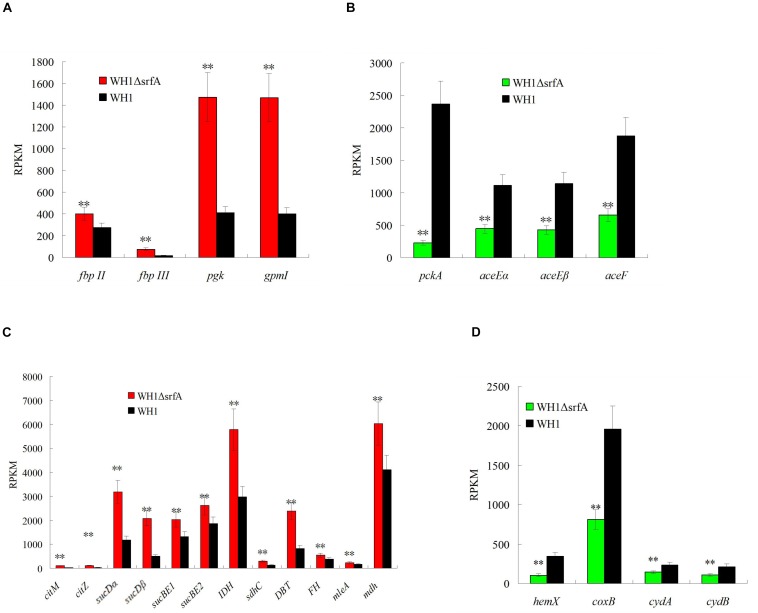
Comparison of the gene transcription involved in EMP, pyruvate dehydrogenation, TCA, and oxidative phosphorylation between Δ*srfA* and WH1. **(A)** EMP. **(B)** Pyruvate dehydrogenation. **(C)** TCA. **(D)** Oxidative phosphorylation. Data were analyzed on the basis of transcriptomes. Double stars indicate significant difference (*p* < 0.01) between Δ*srfA* and WH1.

Mutation of *srfA* led to the cell death in *B. amyloliquefaciens*. For clues to explain this phenomenon, we compared the transcriptomes between Δ*srfA* and WH1, and found that Δ*srfA* showed a significant difference of genes transcription about cellular process, metabolic process, single organism process, and catalytic activity from WH1 (Supplementary Figure S5). Further analysis revealed that carbon metabolism was very different between WH1 and Δ*srfA*. The carbon metabolism was seriously damaged in Δ*srfA*. Compared to WH1, the Embden–Meyerhof pathway (EMP) that catalyzes glucose to pyruvate was enhanced in Δ*srfA*. The key gene transcriptions involved in EMP such as *fbp* (fructose-1,6-bisphosphatase), *pgk* (phosphoglycerate kinase), and *gpmI* (2,3-bisphosphoglycerate-independent phosphoglycerate mutase) were all significantly up-regulated in Δ*srfA* (Figure 2A). Pyruvate is catalyzed to acetyl-CoA and then enters into the citrate cycle (TCA). In this progress, several key gene transcriptions such as *pckA* (phosphoenolpyruvate carboxykinase), *aceE* (pyruvate dehydrogenase E1 component), and *aceF* (pyruvate dehydrogenase E2 component) were significantly down-regulated (Figure 2B), and the key gene transcriptions involved in TCA such as *citM* (citrate transporter), *citZ* (citrate synthase), *sucD* (succinyl-CoA synthetase), *sucB* (2-oxoglutarate dehydrogenase), *IDH* (isocitrate dehydrogenase), *sdhC* (succinate dehydrogenase cytochrome b-556 subunit), *DBT* (branched-chain alpha-keto acid dehydrogenase subunit E2), *FH* (fumarate hydratase), *mleA* (malate dehydrogenase), and *mdh* (malate dehydrogenase) were all significantly up-regulated in Δ*srfA* (Figure 2C). In the TCA cycle, NADH is produced and then enters into the oxidative phosphorylation for production of ATP. In this progress, several key gene transcriptions such as *hemX* (cytochrome c assembly protein), *coxB* (cytochrome c oxidase), *cydA* (cytochrome d ubiquinol oxidase subunit I), and *cydB* (cytochrome d ubiquinol oxidase subunit II) were all significantly down-regulated in Δ*srfA* (Figure 2D). Thereby, the basic carbon metabolism is disordered in Δ*srfA* due to deficiency of surfactin.

### Dissolved O_2_ in Culture Was Not Influenced by Surfactin

Surfactin is a powerful biosurfactant, thus possibly influences the dissolved O_2_ content in culture following with an effect on the cellular carbon and energy metabolism. However, the dissolved O_2_ content in Δ*srfA* was not lower than WH1 (Supplementary Figure S6), implying there is no limitation of dissolved O_2_ in the culture of Δ*srfA*.

### Obstacle to Use Non-preferred Carbon Sources Due to Deficiency of Surfactin

**FIGURE 3:**
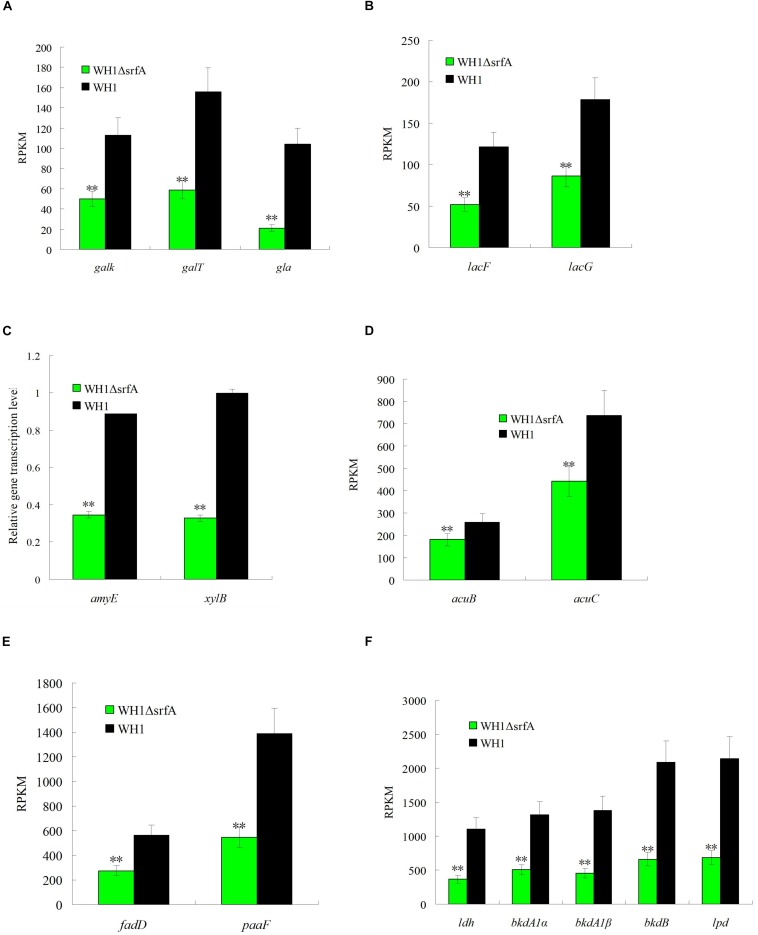
Comparison of the gene transcription involved in utilization of non-preferred carbon sources between Δ*srfA* and WH1. **(A)** Galactose. **(B)** Lactose. **(C)** Starch and xylose. **(D)** Acetoin. **(E)** Fatty acids. **(F)** Branched-chain amino acids. **(A)**, **(B)**, **(D)**, **(E)**, and **(F)** were analyzed on the basis of transcriptomes, and **(C)** was analyzed by qRT-PCR. Double stars indicate significant difference (*p* < 0.01) between Δ*srfA* and WH1.

It was paradoxical that the death of Δ*srfA* began at ∼18 h because the residual nutrients were still enough for growth. Thereby, we further analyzed the ability to utilize non-preferred carbon sources such as galactose, lactose, xylose, fatty acids, amino acids, acetoin, etc. in Δ*srfA*. Compared to WH1, the genes transcription involved in utilization of galactose such as *galK* (galactokinase), *galT* (UDP-glucose-hexose-1-phosphate uridylyltransferase) and *gla* (alpha-galactosidase) were all significantly down-regulated in Δ*srfA* (Figure 3A). The key genes transcription involved in lactose metabolism such as *lacF* (PTS system, lactose-specific IIA component) and *lacG* (6-phospho-beta-galactosidase) were also significantly down-regulated in Δ*srfA* (Figure 3B). Other key genes involved in utilization of non-preferred carbon sources such as *amyE* (α-amylase) for metabolizing starch and *xylB* (Xylulose kinase) for utilizing xylose were both significantly down-regulated in Δ*srfA* (Figure 3C). Acetoin is a product from carbon overflow metabolism, and can be used as a non-preferred carbon source in *B. subtilis*. The results showed the genes transcription involved in utilization of acetoin such as acetoin utilization proteins (*acuB* and *acuC*) were significantly down-regulated in Δ*srfA* (Figure 3D).

In addition to extracellular carbon sources, the key genes transcription involved in utilizing intracellular carbon sources such as fatty acids and branched-chain amino acids were also significantly down-regulated in Δ*srfA.* Compared to WH1, the transcription of genes involved in degradation of fatty acids such as long-chain acyl-CoA synthetase (*ACSL*/*fadD*) and enoyl-CoA hydratase (*paaF*/*echA*) were both significantly down-regulated in Δ*srfA* (Figure 3E). The branched-chain amino acids including Ala, Val, Leu and Ile can be utilized as carbon sources in *B. subtilis*. The transcription of several key genes involved in degradation of branched-chain amino acids such as leucine dehydrogenase (*ldh*), 2-oxoisovalerate dehydrogenase E1 component alpha subunit (*bkdA1*) and E2 component (dihydrolipoyl transacylase) (*bkdB*), and dihydrolipoamide dehydrogenase (*lpd*/*pdhD*), were all significantly down-regulated in Δ*srfA* (Figure 3F). All of these results indicated the ability to use non-preferred carbon sources is weakened in Δ*srfA.*

### High Expression of ccpA in ΔsrfA

**FIGURE 4:**
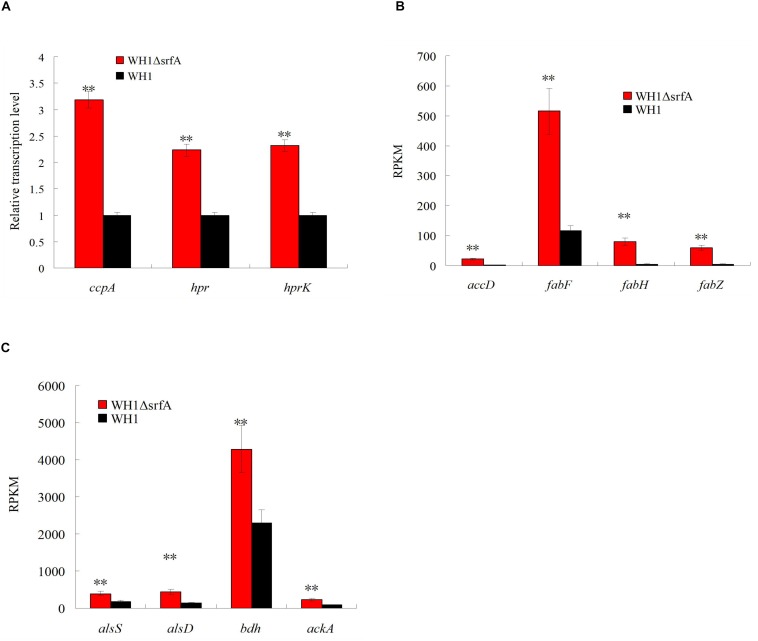
Comparison of the transcription of *ccpA* and the genes controlled by CcpA between Δ*srfA* and WH1. **(A)** Transcription of *ccpA*, *hpr*, and *hprK*. **(B)** Transcription of genes involved in biosynthesis of fatty acids. **(C)** Transcription of genes involved in carbon overflow metabolism. **(A)** was analyzed by qRT-PCR, and **(B)** and **(C)** were analyzed on the basis of transcriptomes. Double stars indicate significant difference (*p* < 0.01) between Δ*srfA* and WH1.

CcpA inhibits utilization of non-preferred carbon source by forming a complex with HPr∼P in *B. subtilis*. Analysis by qRT-PCR, we found the genes transcription of *ccpA*, *hpr* and *hprK* (HPr kinase) were all significantly up-regulated in Δ*srfA* when compared to WH1 (Figure 4A), indicated the CcpA-mediated CCR is enhanced in this strain. CcpA also promotes the genes transcription in many carbon metabolism such as EMP, biosynthesis of fatty acids, carbon overflow. The results showed the genes transcription involved in biosynthesis of fatty acids such as *accD* (acetyl-CoA carboxylase carboxyl transferase), *fabF* (3-oxoacyl-[acyl-carrier-protein] synthase II), *fabH* (3-oxoacyl-[acyl-carrier-protein] synthase III), and *fabZ* (3-hydroxyacyl-[acyl- carrier-protein] dehydratase), were all significantly up-regulated in Δ*srfA* (Figure 4B). The genes transcription involved in carbon overflow metabolism such as *alsS* (acetolactate synthase), *alsD* (acetolactate decarboxylase), *bdh* (2,3-butanediol dehydrogenase) and *ackA* (acetate kinase), were all significantly up-regulated in Δ*srfA* (Figure 4C). These results all suggested that the enhanced *ccpA* transcription improves the genes transcription involved in bio-synthesis of fatty acids, carbon overflow metabolism and EMP (Figure 2A) in Δ*srfA*.

### High Expression of Genes Involved in Chemotaxis and Motility in ΔsrfA

Because of the disability to produce surfactin, Δ*srfA* could not use non-preferred carbon sources after glucose exhaustion. Thereby, the cells searched for a new environment with enough glucose for survival by up-regulation of the genes transcription involved in chemotaxis such as methyl-accepting chemotaxis protein (*mcpA*, *mcpB*, *mcpC*, *tlpA*, *tlpB*, *yfmS*), chemotaxis protein (*cheC*, *cheV*, and *cheW*), chemotaxis protein methyltransferase (*cheR*), sensor histidine kinase (*cheA*), chemotaxis response regulator (*cheY*) and chemoreceptor glutamine deamidase (*cheD*) in Δ*srfA* (Supplementary Figure S7A). Also, the transcription of genes involved in motility such as swarming motility protein (*swrAA*, *swrB*, and *swrC*), flagellar motor protein (*motA*, *motB*, *motP*, and *motS*), flagellar capping protein (*fliD*), flagellar assembly protein (*fliS*, *fliT*, and *fliW*), flagellar motor switch protein (*fliM*, *fliN*), flagellar biosynthesis protein (*fliP*, *fliQ*, *fliR*, and *fliZ*), flagellar hook-basal body protein (*fliE*), flagellar basal body-associated protein (*fliL*), and flagellar basal-body rod protein (*flhO*), were all significantly up-regulated in Δ*srfA* (Supplementary Figure S7B).

### Weakened Enzyme Activities to Utilize Non-preferred Carbon Sources in ΔsrfA

**FIGURE 5:**
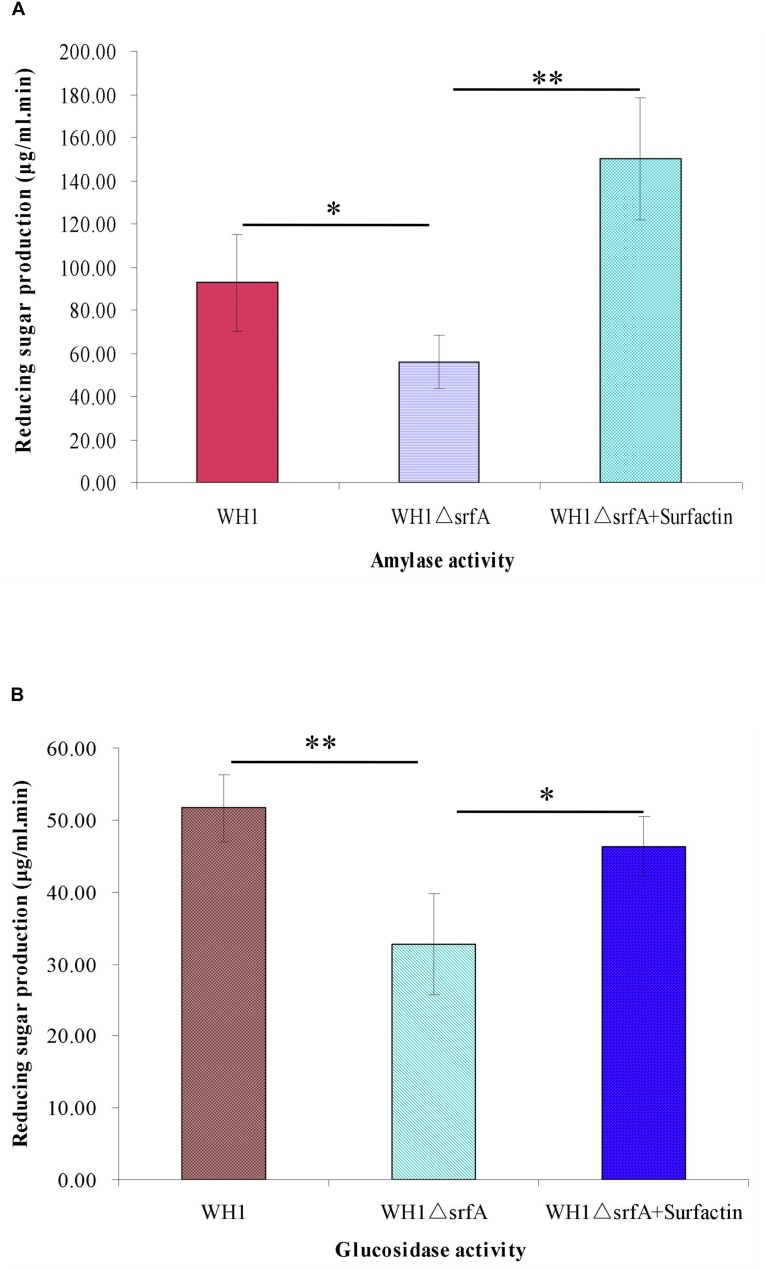
Comparison of the activity of α-amylase and glucanase between WH1 and Δ*srfA*. **(A)** Amylase activity. **(B)** Glucanase activity. The activity of α-amylase and glucosidase was significantly weakened in Δ*srfA* but could be dramatically improved by addition with surfactin. Single star and double stars indicate significant (*p* < 0.05) and very significant (*p* < 0.01) difference between two groups, respectively.

Many enzymes for utilizing non-preferred carbon sources such as amylase for utilizing starch and glucosidase for utilizing glucan, are controlled by the CcpA-mediated CCR in *B. subtilis*. We determined the activity of α-amylase and glucosidase in WH1 and Δ*srfA* respectively, and found the α-amylase activity was significantly weakened in Δ*srfA* when compared to WH1 (Figure 5A). Similarly, the glucosidase activity of Δ*srfA* was also significantly lower than WH1 (Figure 5B). However, addition with surfactin could dramatically increase the activity both of α-amylase (Figure 5A) and glucosidase (Figure 5B) in Δ*srfA*. The results further verified that Δ*srfA* could not effectively utilize the non-preferred carbon sources such as starch and glucan after glucose exhaustion due to deficiency of surfactin.

### Addition With Glucose Dramatically Restoring Defective Growth in ΔsrfA

**FIGURE 6:**
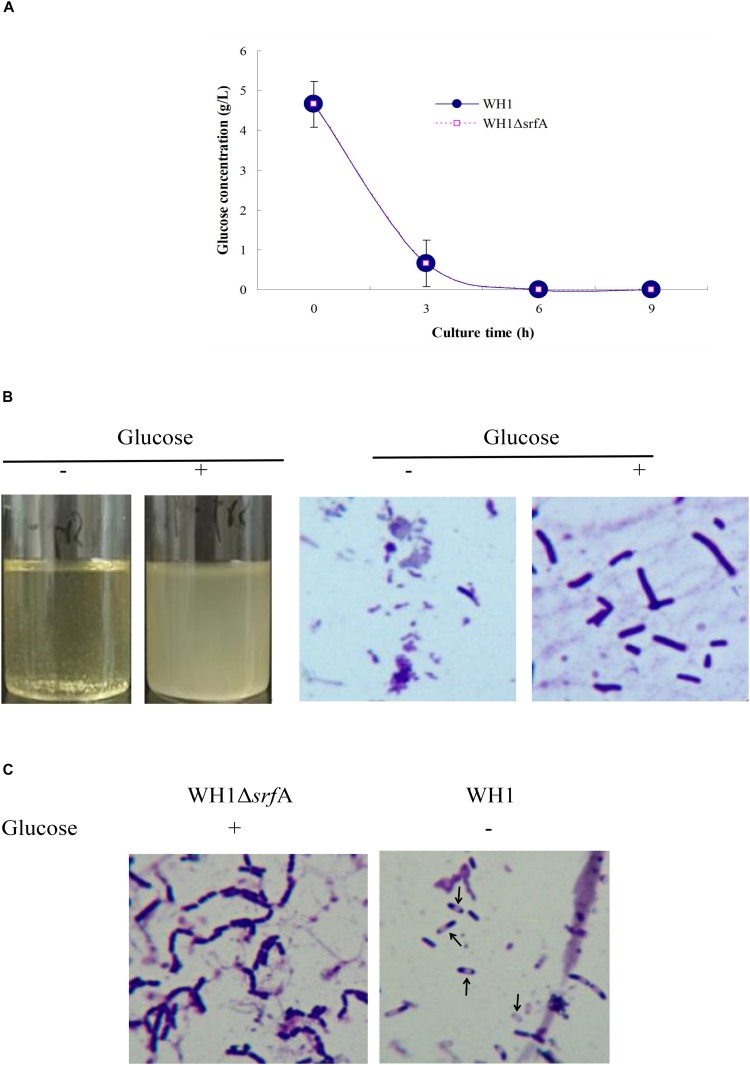
Addition with glucose restored the growth of Δ*srfA* in LB medium. **(A)** Residual glucose in LB medium. Both WH1 and Δ*srfA* showed a similar profile to utilize the glucose in LB medium. **(B)** Addition with glucose (4 g/L) dramatically restored the growth of Δ*srfA* in LB medium (magnification 1000×). **(C)** Addition with glucose could not restore the sporulation in Δ*srfA* (magnification 1000×). Spores are indicated by arrows.

The glucose concentration was about 4.66 g/L in the fresh LB medium. Δ*srfA* showed a similar profile to utilize the glucose in LB medium like WH1 in 6 h (Figure 6A). We added glucose (4 g/L) into the fresh LB medium, and found addition with glucose could dramatically alleviate the cell death in Δ*srfA* due to deficiency of surfactin. Under microscope, the bacterial cells grew well in the LB medium supplemented with glucose (Figure 6B). However, addition with glucose could not restore the sporulation in Δ*srfA* (Figure 6C). We further used the modified M9 medium containing a mixed carbon sources (glucose and xylose) to study whether Δ*srfA* could utilize xylose after glucose exhaustion. The result showed Δ*srfA* grew weakly in the M9 medium with mixed sugars when compared to WH1. After glucose exhaustion, WH1 could use xylose for growth while Δ*srfA* could not due to lack of surfactin (Supplementary Figure S8).

### Mutation of srfA Had Obviously an Effect on CcpA Dependent CCR

**FIGURE 7:**
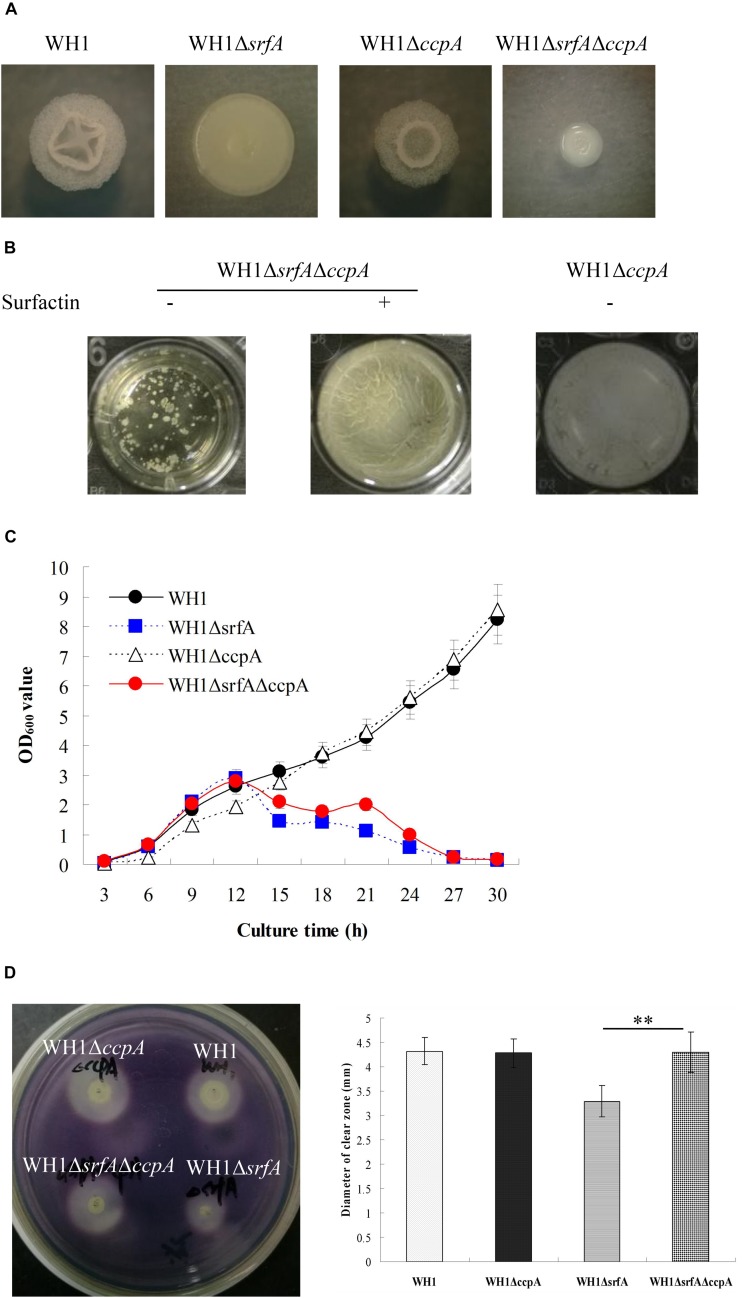
Mutation of *srfA* had obviously an effect on CcpA-dependent CCR. **(A)** Colony morphology of *ccpA*-null mutant strains. **(B)** Addition with surfactin restored the biofilm formation in Δ*srfA*Δ*ccpA*. **(C)** Growth curves of *ccpA*-null mutant strains in LB medium. Knockout of *ccpA* could partially restore the growth of Δ*srfA* in LB medium. **(D)** The α-amylase activity of wild type and mutants on LB agar plates supplemented with starch. The α-amylase activity was weakened in Δ*srfA* compared to WH1, but could be restored by further knockout of *ccpA* in Δ*srfA*. Double stars indicate significant difference (*p* < 0.01) between two groups.

CcpA is known to repress the genes transcription involved in utilization of non-preferred carbon sources. To know whether CcpA was required for surfactin to relieve CCR, we deleted the *ccpA* gene in WH1 and Δ*srfA* respectively (Supplementary Figures S9A,B). The colony morphology of Δ*ccpA* was slightly different from WH1, but Δ*srfA*Δ*ccpA* was very different from WH1, Δ*srfA* and Δ*ccpA* (Figure 7A). Δ*srfA*Δ*ccpA* formed a dispersed and fragile float biofilm that was also very different from WH1, Δ*srfA* and Δ*ccpA*. After addition with surfactin, the defective biofilm could also be restored in Δ*srfA*Δ*ccpA* (Figure 7B), suggested CcpA is not required for the role of surfactin in regulating biofilm formation.

Growth curves showed that knockout of *ccpA* alone did not influence the bacterial growth in LB medium, but Δ*srfA*Δ*ccpA* grew better than Δ*srfA* after 12 h (Figure 7C), suggesting that further knockout of *ccpA* is favorable for restoring the growth of Δ*srfA* in LB medium. The *amyE* gene encoding extracellular α-amylase is subject to the CcpA-mediated CCR in *B. subtilis* (Fujita, 2009). Here, the α-amylase activity was weakened in Δ*srfA* compared to WH1 (Figure 7D), consistent with the results in Figure 5A. However, the weakened enzyme activity could be restored by knockout of *ccpA* in Δ*srfA* (Figure 7D). These results suggested that mutation of *srfA* might obviously affect the CcpA-mediated CCR in *B. amyloliquefaciens*.

## Discussion

*B. amyloliquefaciens* is a close relative to and shares many characteristics with *B. subtilis*. Previously, we isolated a strain of *B. amyloliquefaciens* WH1 with production of surfactin, iturin and fengycin that are potential to be used as anti-fungal pesticides (Qi et al., 2010), and delivery system for oral delivery of protein/peptide drugs (Xing et al., 2018). For high production of single lipopeptide, we mutated the lipopeptides gene including *srfA*, *ituB* and *fenA* in WH1, respectively. As expected, mutation of these genes disrupted the lipopeptides production. Differently, mutation of *srfA* led to a defective biofilm formation and sporulation, suggesting surfactin regulates the QS response in *B. amyloliquefaciens*. This is agreement with the previous report that surfactin is a QS signal molecule in *B. subtilis* (López et al., 2009b). Unexpectedly, mutation of *srfA* also led to a severely defective growth that is not reported previously. Apparently, the death of Δ*srfA* was not attributed to nutrients limitation because WH1 grew well during this period. In fact, the cells death was due to the deficiency of surfactin because compensation with surfactin could restore the bacterial growth in Δ*srfA*. This was further verified by knockout of *comA*, which is essential for the *srfA* transcription in *B. subtilis* (Gonźalez-Pastor, 2011). Δ*comA* showed a defective growth like Δ*srfA* that could also be partially alleviated by addition with surfactin.

Surfactin has a structure consisting of a cyclic heptapeptide headgroup (Glu-Leu-D-Leu-Val-Asp-D-Leu-Leu) linked to a C13-15 β-hydroxy fatty acid by lactone bond (Zhang et al., 2017). This structure endows surfactin with a powerful biosurfactant activity by its amphiphilic nature, a polar amino acid head and a hydrocarbon chain (Carrillo et al., 2003). Thereby, surfactin is considered as a powerful biosurfactant that can cause pores on membrane by its biosurfactant activity (Shaligram and Singhal, 2010). Besides for surfactin, other antibiotics such as bacitracin, amphotericin B and nystatin could also partially restore the growth and biofilm formation in Δ*srfA*. Possibly, these antibiotics seem to have some common properties with surfactin, which can substitute surfactin signaling even they are with different structures.

Addition with surfactin could also restore the biofilm formation and sporulation in Δ*srfA*, consistent with the previous reports (López et al., 2009a, b, 2010; Gonźalez-Pastor, 2011). This raises another question that the death of cells is due to the defective biofilm formation and sporulation in Δ*srfA*? Our data showed that besides for surfactin, glucose could also restore the growth of Δ*srfA*; however, addition with glucose could not restore the sporulation in this strain. Therefore, it seems that the disability to sporulate is not the reason for the cell death of Δ*srfA*. Actually, it likes that the cells had a defective carbon metabolism in Δ*srfA* because the nutrients in medium were enough for growth but they still needed additional glucose for survival. This conception was further strengthened by the data of transcriptomes that revealed a severely defective carbon metabolism in Δ*srfA*. As a feedback of glucose limitation, the EMP pathway was significantly enhanced in Δ*srfA* (Fujita, 2009). In the progress of catalyzing pyruvate to acetyl-CoA, several key genes transcription were significantly down-regulated due to lack of pyruvate. Thereby, there was enough acetyl-CoA in cells. As a feedback to acetyl-CoA limitation, several key genes transcription in TCA were up-regulated in Δ*srfA*. In the TCA cycle, NADH is produced, then enters into the oxidative phosphorylation for producing ATP (Fujita, 2009). Due to lack of NADH, several key genes transcription involved in this progress were significantly down-regulated in Δ*srfA*. All of these results suggested that the basic carbon metabolism is disordered in Δ*srfA*.

What is the reason for the disordered carbon metabolism in Δ*srfA*? Because surfactin is one of the most effective biosurfactants discovered so far (Shaligram and Singhal, 2010), we deduced that surfactin might influence the dissolved O_2_ content in culture. Without enough O_2_, the carbon and energy metabolism will be limited in cells (Zhu et al., 2015). However, the dissolved O_2_ content in Δ*srfA* was not lower than WH1, suggested the disordered carbon metabolism was not attributed to the limitation of dissolved O_2_ in Δ*srfA*. In fact, the ability to utilize non-preferred carbon sources were defective in Δ*srfA* because the transcription of genes involved in utilizing galactose, lactose, xylose, starch, acetoin, fatty acids and branched-chain amino acids were all significantly down-regulated in this strain. This was also consistent with the activity of α-amylase and glucosidase which were both significantly decreased in Δ*srfA* but could be restored by addition with surfactin. Thereby, Δ*srfA* could not utilize non-preferred carbon sources after glucose exhaustion due to deficiency of surfactin. This was further verified by the result that addition with glucose could dramatically restore the growth of Δ*srfA*. Additionally, the dissolved O_2_ content also supported that Δ*srfA* is defective to use non-preferred carbon sources. After glucose was rapidly exhausted, Δ*srfA* could not use non-preferred carbon sources hence there was no carbon source to be used for consuming O_2_ (Zhu et al., 2015). As a result, the dissolved O_2_ content was increased in Δ*srfA*.

Generally, the ability to use non-preferred carbon sources are controlled by CcpA in *Bacillus* spp. such as *B. subtilis* (Gorke and Stulke, 2008; Fujita, 2009). Thereby, the disability to utilize non-preferred carbon sources might be due to the enhanced repression mediated by CcpA in Δ*srfA*. Indeed, the genes transcription of *ccpA*, *hpr* and *hprK* (Müller et al., 2006; Schumacher et al., 2011), were all significantly up-regulated in Δ*srfA* due to deficiency of surfactin. Addition to repression, CcpA also promotes many genes transcription involved in carbon metabolism (Fujita, 2009; Yang et al., 2017). In Δ*srfA*, several genes transcription involved in EMP, carbon overflow metabolism and biosynthesis of fatty acids were all significantly up-regulated. These results also indirectly confirmed that the CcpA-mediated CCR is enhanced in Δ*srfA*. Nevertheless, the growth of Δ*srfA* could be partially restored by further knockout of *ccpA.* Consistently, the extracellular α-amylase activity was weakened in Δ*srfA* but could be restored by further knockout of *ccpA*. Thereby, the *srfA* deletion has obviously an effect on the CcpA dependent CCR in *B. amyloliquefaciens*.

It seems that the lack of surfactin causes not only an inability to use non-preferred carbon sources but additionally lysis of the cells. It might be expected that a deficit of nutrients would first only stop growth for a while before lysis occurs due to starvation. The *srfA* mutant does not stop only growth but starts to lyse immediately after 12 h. Thereby, in order to survival the cells of Δ*srfA* would search for a new environment with enough glucose. Indeed, many genes transcription involved in chemotaxis and motility (Mirouze and Dubnau, 2013; van Gestel et al., 2015), were significantly up-regulated in Δ*srfA*. The result is consistent with the previous report that after nutrients limitation, *B. subtilis* could synthesize a complex motility and chemotaxis system searching for nutrients in the environment (Hamoen et al., 2003).

Although both of surfactin and glucose could restore the growth in Δ*srfA*, it was only surfactin rather than glucose with an ability to restore the sporulation in this strain. Addition with glucose only supplemented a preferred carbon source for Δ*srfA* while addition with surfactin could trigger a signaling pathway to relieve CCR in this mutant strain. The signaling triggered by surfactin could not only relieve CCR to utilize non-preferred carbon sources, but also regulate the QS response such as biofilm formation and sporulation in *B. amyloliquefaciens* (López et al., 2009a, 2010; Vlamakis et al., 2013; Xu et al., 2013; Zeriouh et al., 2014).

Surfactin is a QS molecule in *B. subtilis* (López et al., 2010), by which the cells undergo several adaptive responses to nutrients depletion and high cell density in the environment (Ohsawa et al., 2006). In *B. subtilis*, prolonged nutritional stress can result in the development of QS response such as biofilm formation and sporulation (Hamoen et al., 2003). Here, the preferred carbon sources such as glucose were exhausted that acts as a nutritional stress to induce production of surfactin triggering the QS response in *B. amyloliquefaciens*. Possibly, the signaling triggered by surfactin has either a direct or an indirect effect on HPrSer_46_P dephosphorylation by HPrK/P (Mijakovic et al., 2002; Poncet et al., 2004; Fujita, 2009). For example, it can cause low intracellular Pi levels or it reduces somehow HPrK/P phospho-phosphorylase activity, but further studies are needed for verifying this conception.

By surfactin, QS tightly links with carbon metabolism to counter nutrients limitation in the natural environment. Generally, the available carbon sources are composed of diverse sugars in the complex environment (Li et al., 2014), and the preferred carbon sources (e.g., glucose) are preferentially utilized by cells for a fast growth (Müller et al., 2006). After exhaustion, the cells produce surfactin to relieve CCR utilizing the non-preferred sugars. Thereby, as a signal molecule to regulate QS, surfactin also directly or indirectly relieves CCR to utilize non-preferred carbon sources countering nutrients limitation (e.g., glucose deprivation) in the environment. In **conclusion**, our findings provide the first evidence that the QS signal molecule of surfactin is also involved in reliving the CcpA-mediated CCR in *B. amyloliquefaciens*.

## Data Availability Statement

The raw data were uploaded to SRA database and the BioProject ID is PRJNA560470.

## Author Contributions

GQ and XZ conceived, designed, and coordinated the study, and drafted the manuscript. BC, JW, and JD constructed mutant strains, determined phenotype, and performed biochemical studies. BC determined lipopeptides. JD and JW analyzed transcriptomes. All authors analyzed the data, provided critical input into the final manuscript, and approved the final version.

## Conflict of Interest

The authors declare that the research was conducted in the absence of any commercial or financial relationships that could be construed as a potential conflict of interest.
