# Supplementary material for: Surfactin: A Quorum-Sensing Signal Molecule to Relieve CCR in Bacillus amyloliquefaciens
Source: Front Microbiol. 2020 Apr 30;11:631. doi: 10.3389/fmicb.2020.00631 (PMC7203447; doi:10.3389/fmicb.2020.00631)
Supplement: Supplementary file 1 [file Data_Sheet_1.docx]

**Table** **S1 Bacterial strains and plasmids used in this study**

| Strains/ plasmids | Characteristics | Source |
| --- | --- | --- |
| *Escherichia coli* DH5α | Used for construction and amplification of plasmids | Stored in this lab |
| *Bacillus amyloliquefaciens* WH1 | Wild-type | Stored in this lab |
| T2(2)-ori | *E. coli*-*Bacillus* shuttle vector, ori_pUC_/ori_ts_, temperature-sensitive, Kan^r^ | Stored in this lab |
| WH1Δ*srfA* | *srfA* mutant strain | This study |
| WH1Δ*ituB* | *ituB* mutant strain | This study |
| WH1Δ*fenA* | *fenA* mutant strain | This study |
| WH1Δ*comA* | *comA* knockout strain | This study |
| WH1Δ*ccpA* | *ccpA* knockout strain | This study |
| WH1Δ*srfA*Δ*ccpA* | *srfA* mutant and *ccpA* knockout strain | This study |

ori_ts_, temperature-sensitive *Bacillus* origin of replication; Kan^r^ , Kanamycin resistance.

**Table S2 Primers used in this study**

| Name | Sequence 5′→3′ | Purpose |
| --- | --- | --- |
| *srfA*-LF | CG**GGATCC**GAATCTTTTTGAAGCGCTCT | Amplifying L arm of *srfA* |
| *srfA*-LR | ATATAAAGC ATGTGTGCGCCTCCCCTTTT |  |
| *srfA*-RF | CGCACACATGCTTTATATCGTGCCGAAAA | Amplifying R arm of *srfA* |
| *srfA*-RR | GC**TCTAGA** CGGACTTTCACCTGATCATC |  |
| *srfA*-single  -crossover LF | TTATGCCGATTTTGGACGCCAT | Verifying single crossover of *srfA* |
| *srfA*-single  -crossover RR | TGCCCTGCTGATCCGGCCGT |  |
| *fenA*-LF | CG**GGATCC**CTTCGGAGCCATTTGATATA | Amplifying L arm of *fenA* |
| *fenA*-LR | CTTGCTGGCTTTCATAAAAAGGTGTGTGG |  |
| *fenA*-RF | TTTATGAAAGCCAGCAAGCCTGATCTCCG | Amplifying R arm of *fenA* |
| *fenA*-RR | GC**TCTAGA**TGGCGAGTTTCTCATTTGA |  |
| *fenA*-single  -crossover LF | GTTTTGCGCCGTCTTATTCT | Verifying single crossover of *fenA* |
| *fenA*-single  -crossover RR | AGGGAGTCGAAGTCAGAAAT |  |
| *ituB*-LF | CG**GGATCC**TGCTCTAGTGAAACAACTGT | Amplifying L arm of *ituB* |
| *ituB*-LR | TTGTTCCGCTCACGGGGGCAGCGGCTGTC |  |
| *ituB*-RF | GCCCCCGTGAGCGGAACAAAAGTTTCGAGT | Amplifying R arm of *ituB* |
| *ituB*-RR | GC**TCTAGA**TGACGAATCTGCTCGCTTAT |  |
| *ituB*-single  -crossover LF | AGCATCTGCAGCAGCCGTT | Verifying single crossover of *ituB* |
| *ituB*-single  -crossover RR | CAGCTGGAGCACAAGACGAT |  |
| *comA*-LF | CG**GGATCC**ATCATTTTTATCCTGCTGAC | Amplifying L arm of *comA* |
| *comA*-LR | GGAGGAAACCATCAAGAAGGGAGAA |  |
| *comA*-RF | CCTTCAAGAT GGTTTCCTCC CTTTT | Amplifying R arm of *comA* |
| *comA*-RR | GC**TCTAGA**TTAAGAAAGAGGAGCCTTGC |  |
| *comA*-single  -crossover LF | TTCAAAAGGCTCGACACTCG | Verifying single crossover of *comA* |
| *comA*-single  -crossover RR | GCTTGCGACGAAAAGGAGCT |  |
| *ccpA*-LF | CGGGATCCCTGTGTCTTTCTTCCATTGC | Amplifying L arm of *ccpA* |
| *ccpA*-LR | CATTGACCGAAACTGATGAACAAAGAGCC |  |
| *ccpA*-RF | CATCAGTTTCGGTCAATGGCTTCCAATAC | Amplifying R arm of *ccpA* |
| *ccpA*-RR | GCTCTAGATGAAACAGGAAACTCACTTG |  |
| *ccpA*-single  -crossover LF | GGTATGCGGATCATTTAAGT | Verifying single crossover of *ccpA* |
| *ccpA*-single  -crossover RR | CAAGGAAACGACCAAATCAT |  |

Restriction sites of *Bam*H I and *Xba* I are highlighted in bold. Underline stands for the overlap region for Splicing by Overlapping Extension PCR (SOE-PCR).

**Table S3 Primers used in qRT-PCR**

| Name | Sequence 5′→3′ |
| --- | --- |
| 16s-qF | CTGCCTGTAAGACTGGGATAAC |
| 16s-qR | CATCTGTAAGTGGTAGCCGAAG |
| *amyE*-qF | TCGCGGGAGTGCTTTATTT |
| *amyE*-qR | TTTCCATTCGGGTTCGAGAG |
| *xylB*-qF | GTGAGATTCCGCATGACTGG |
| *xylB*-qR | CAAATCTCATCGCTCCACTC |
| *ccpA*-qF | GGACCGATGCAAGAGCCGAT |
| *ccpA*-qR | CGCCTTCCGCTACGAATTGC |
| *hpr*-qF | GAAGAACAGGACGGTTTTGA |
| *hpr*-qR | ACTCCCATATCGCCGTTTTG |
| *hprK*-qF | TACGACCGTCTCGGACTTGA |
| *hprK*-qR | CAACTTCGATAATAACGGCG |

**Figure S1**

(A) (B)

M 1 2 3 4


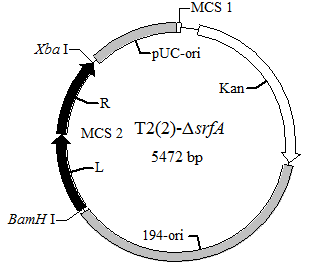


2000

1000


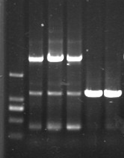


(C) (D)

M 1 2 M 1 2 3 4

2000

1000

2000

1000


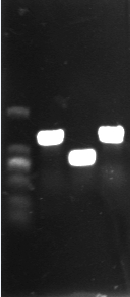

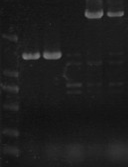


**Fig. S1 Construction of lipopeptides gene mutant strains. (A):** Schematic diagram of the recombinant plasmid for mutation of *srfA* based on T2(2) plasmid. The vector contained temperature-sensitive replicon from *B. subtilis* (194-Ori), kanamycin-resistant gene (Kan), and the homologous arm A and B for homologous recombination. **(B):** Verification of *srfA*-mutated strain (Δ*srfA*) by PCR. Lane 1-3 were PCR products from WH1, while lane 4 was PCR product from the strain with double crossover for mutation of *srfA*. **(C):** Verification of *ituB*-mutated strain (Δ*ituB*) by PCR. Lane 1 was PCR product from WH1 while lane 2 was PCR product from the strain with double crossover for mutation of *ituB*. **(D):** Verification of *fenA*-mutated strain (Δ*fenA*) by PCR. Lane 1 & 2 were PCR products from the strain with double crossover for mutation of *fenA*, while lane 4 was PCR product from WH1. Arrows indicate the objective DNA bands. M: DNA marker.

**Figure S2**

(A)


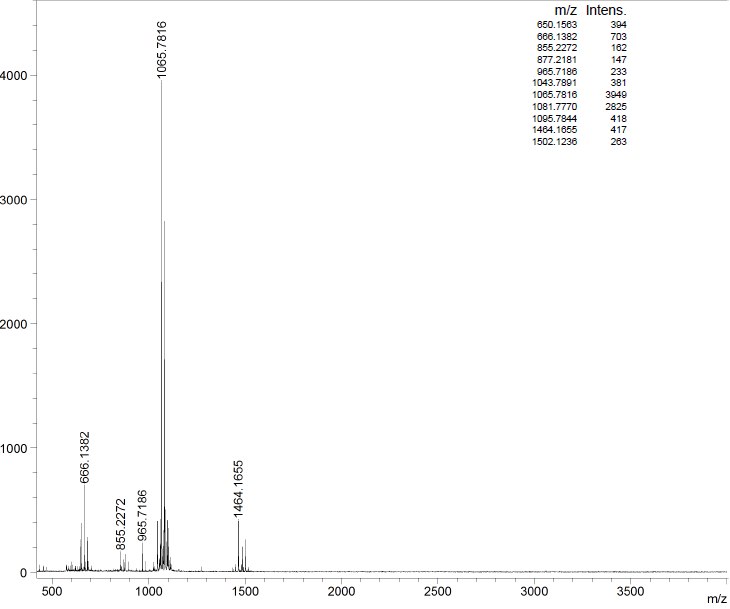


WH1


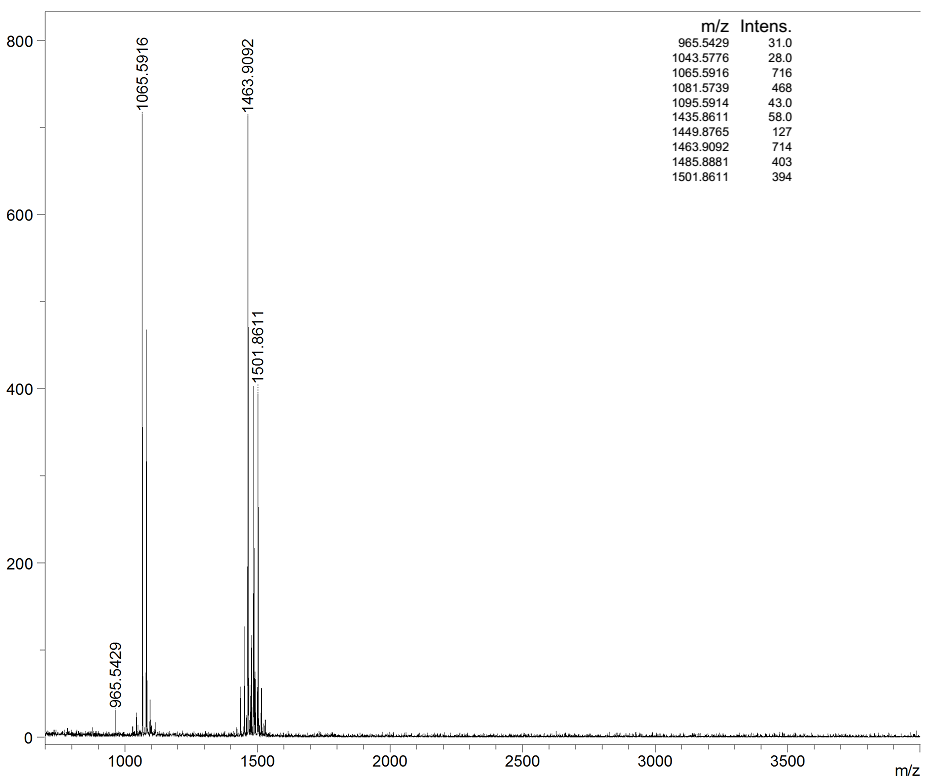


Δ*srfA*


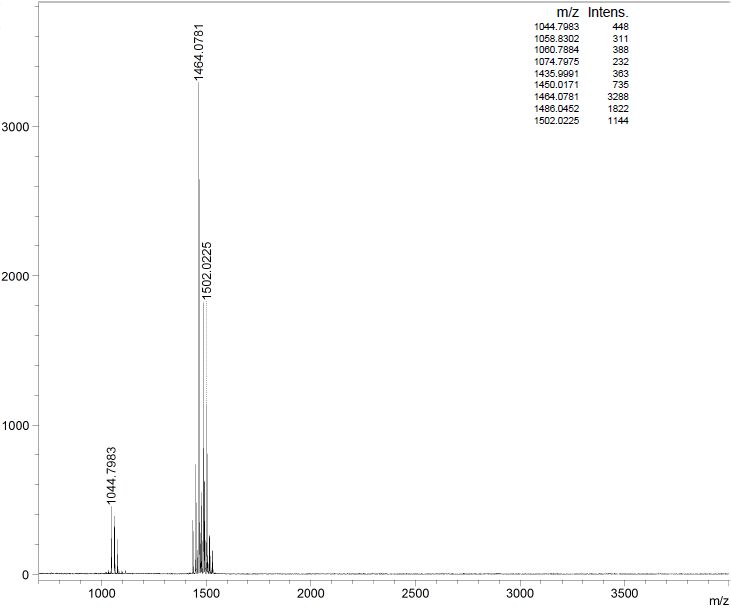


Δ*ituB*


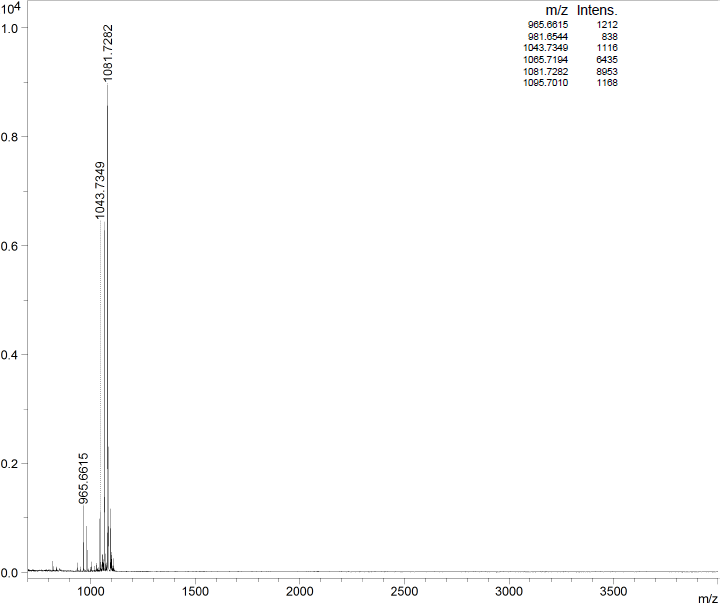


Δ*fenA*

(B)

WH1 Δ*srfA* Δ*ituB*  Δ*fenA*


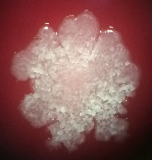

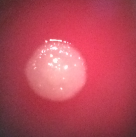

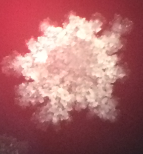

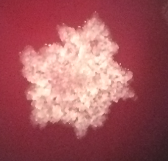


Front


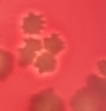

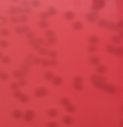

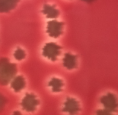

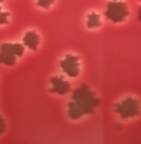


Back

(C)

WH1 Δ*srfA* Δ*ituB* Δ*fenA*


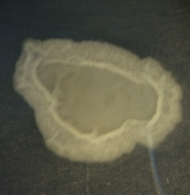

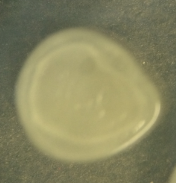

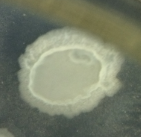

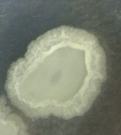


(D)

WH1 Δ*srfA* Δ*ituB* Δ*fenA*


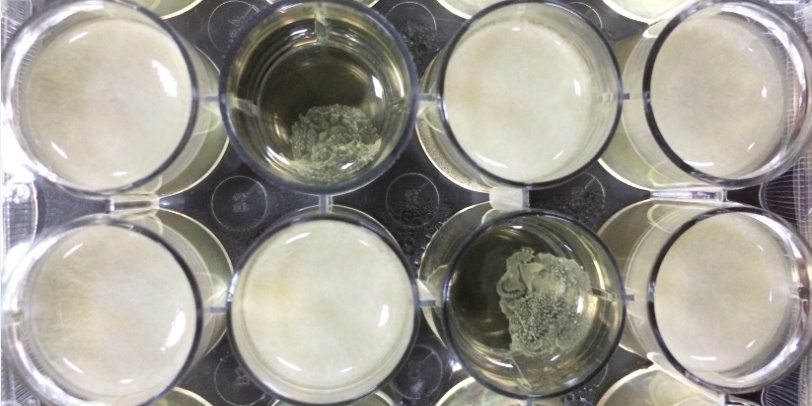

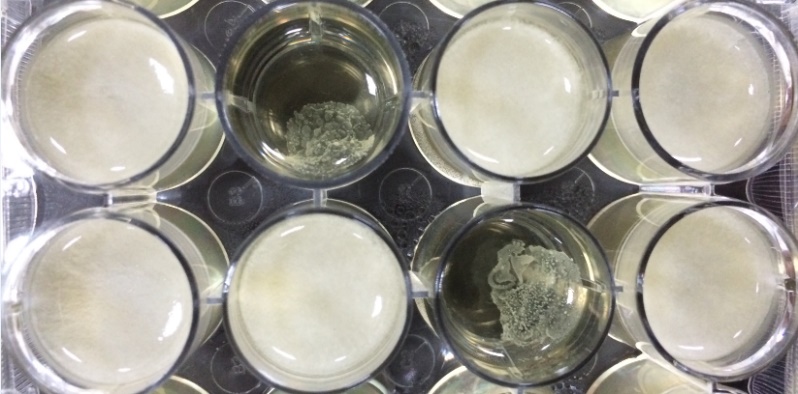

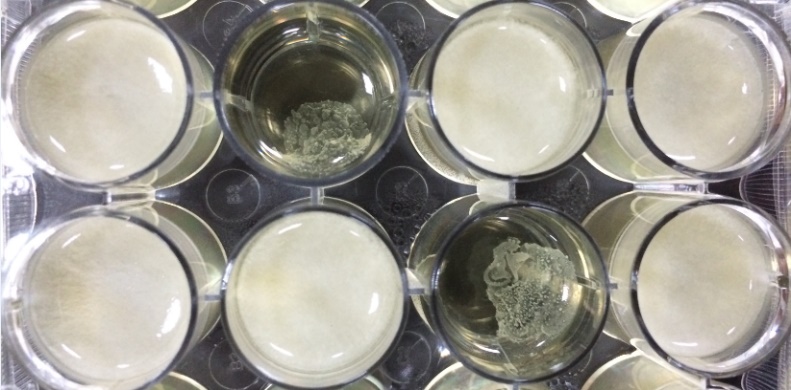

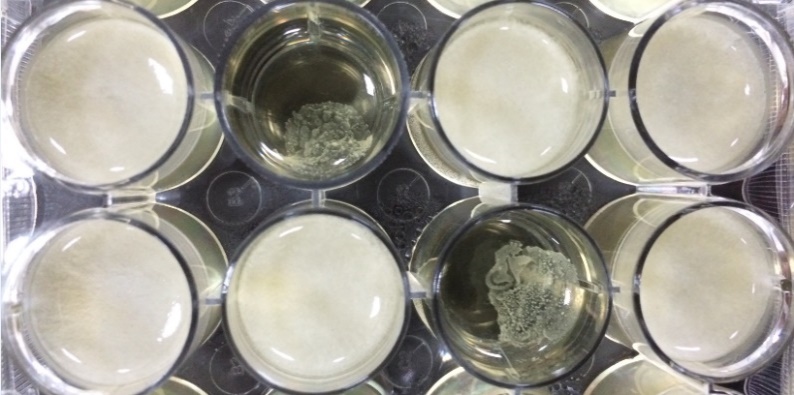


**Fig. S2 Phenotype and lipopeptides production of mutant strains.** **(A):** MALDI-TOF analysis of lipopeptides produced by the wild-type WH1 and the mutants of Δ*srfA*, Δ*ituB* and Δ*fenA*. WH1: surfactin (1044), iturin (1081, 1095), and fengycin (1464, 1502); Δ*srfA*: iturin (1043, 1081, 1095) and fengycin (1435, 1449, 1463, 1501); Δ*ituB*: surfactin (1044) and fengycin (1435, 1464, 1502); Δ*fenA*: surfactin (981) and iturin (1043, 1081). **(B):** Hemolytic activity of different strains on blood agar plates. **(C):** Colony morphology of WH1, and the mutant strains of Δ*srfA*, Δ*ituB* and Δ*fenA*. **(D):** Biofilm of WH1, and the mutant strains of Δ*srfA*, Δ*ituB* and Δ*fenA*.

**Figure S3**

- 20 μg/ml 40 μg/ml 60 μg/ml 80 μg/ml

**
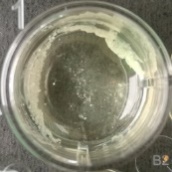

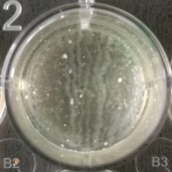

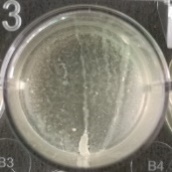

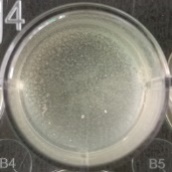

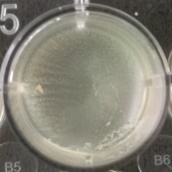
**

Surfactin

**
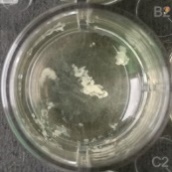

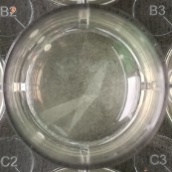

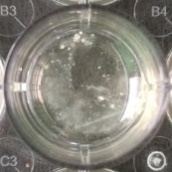

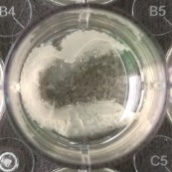

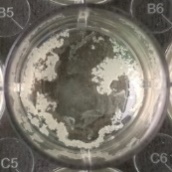
**

Bacitracin

**
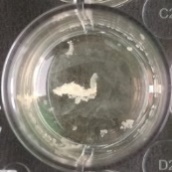

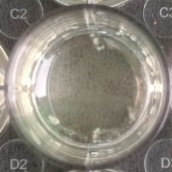

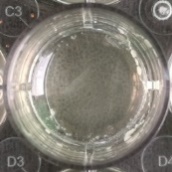

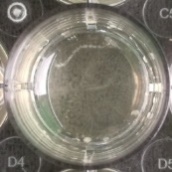

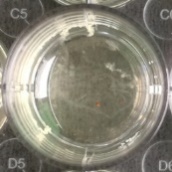
**

Amphotericin B

**
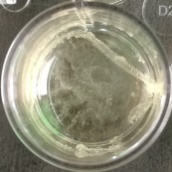

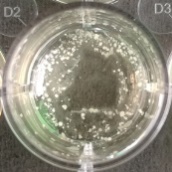

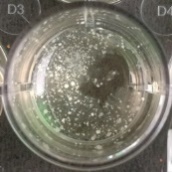

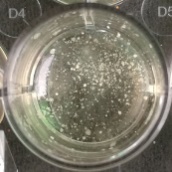

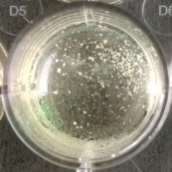
**

Nystatin

**Fig. S3 Addition with antibiotics restored the biofilm formation in Δ*srfA*.** Addition with surfactin could restore the biofilm formation in Δ*srfA*. Except for Amphotericin B, Bacitracin and Nystatin could also partially restore the biofilm formation in Δ*srfA*, but were weaker than surfactin.

**Figure S4**

(A)

WH1 Δ*srf*A Δ*comA*


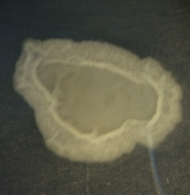

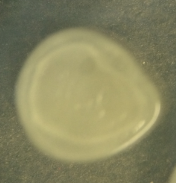

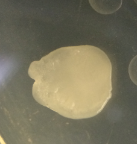


(B)

WH1 Δ*comA*

Surfactin + -


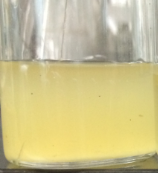

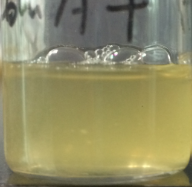

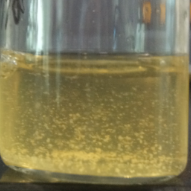


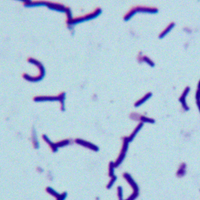

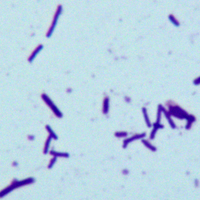

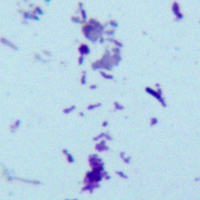


**Fig. S4 Δ*comA*. (A):** Colony morphology of Δ*comA*. **(B):** Effect of surfactin on the growth and survival of Δ*comA* (Magnification 1000×).

**Figure S5**

(A)


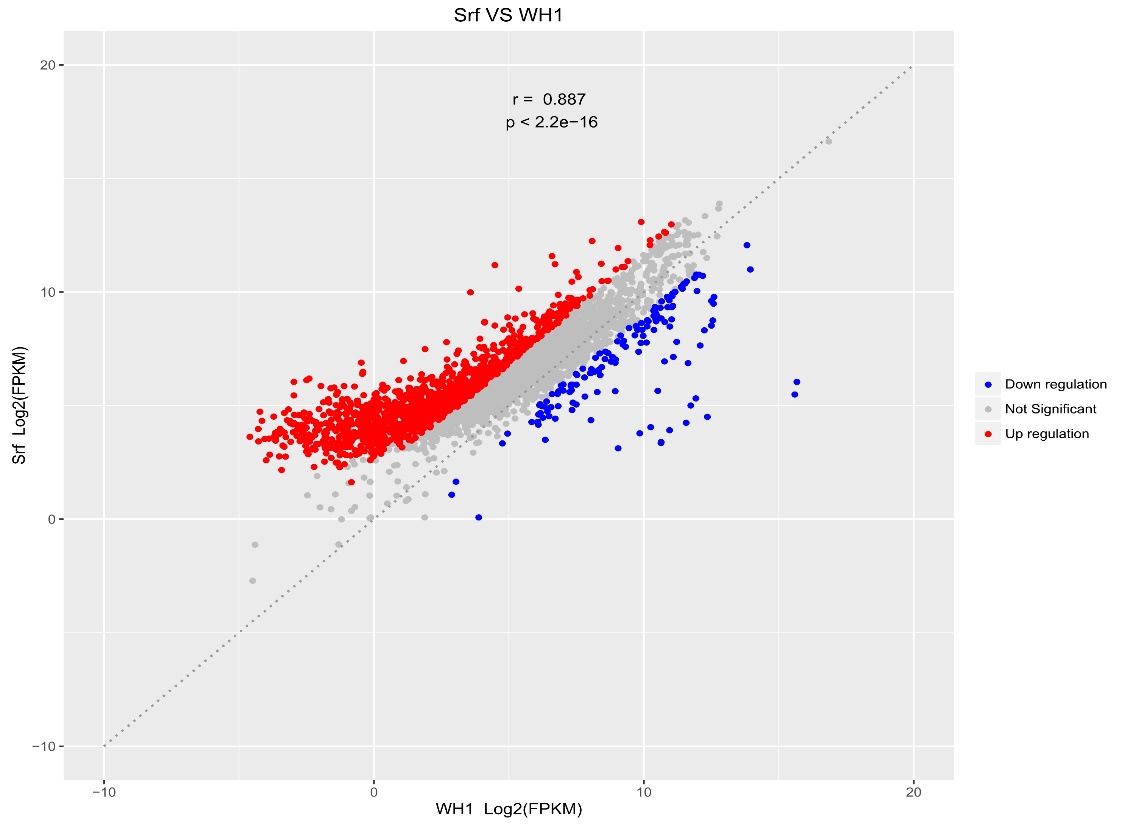


(B)


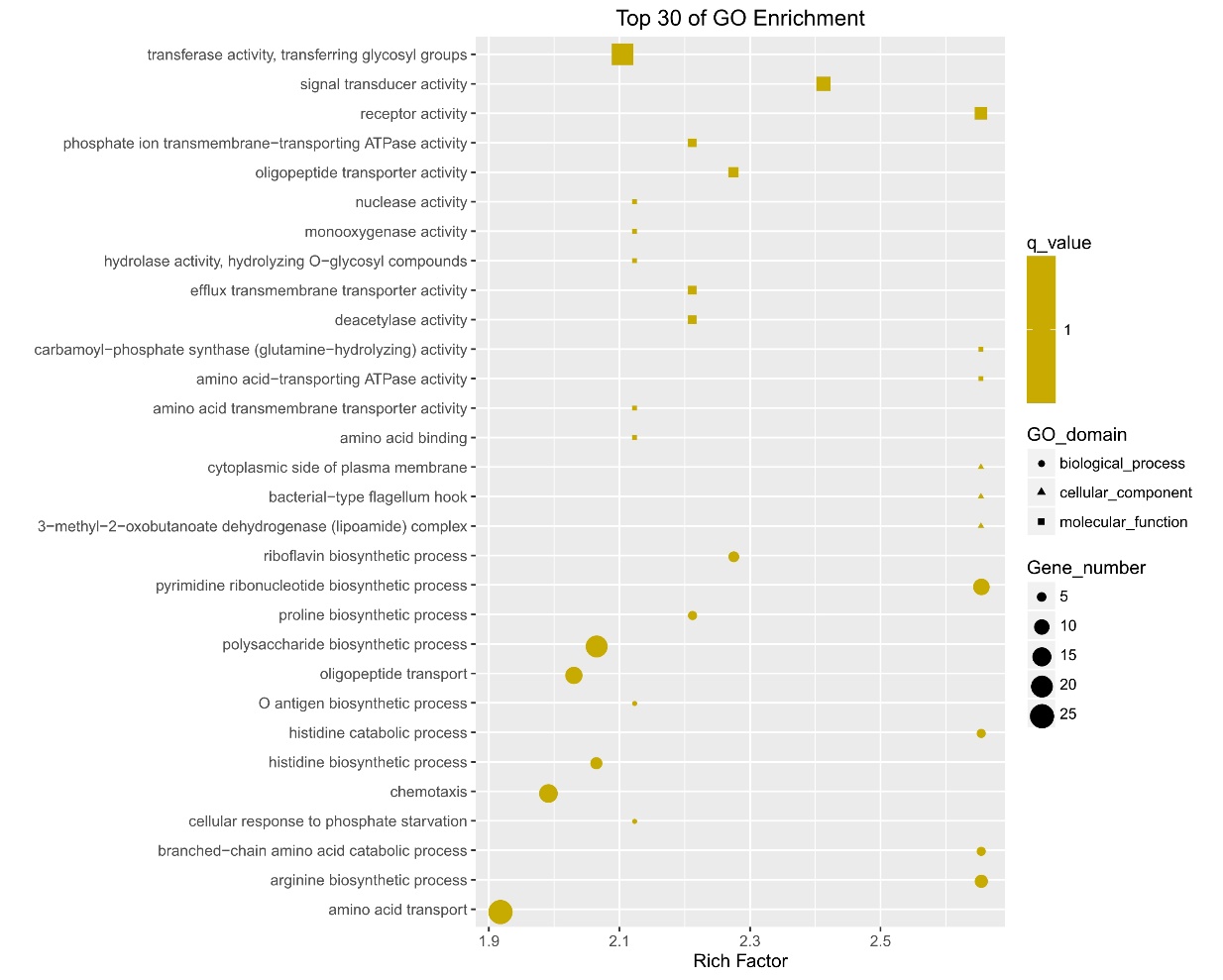


(C)


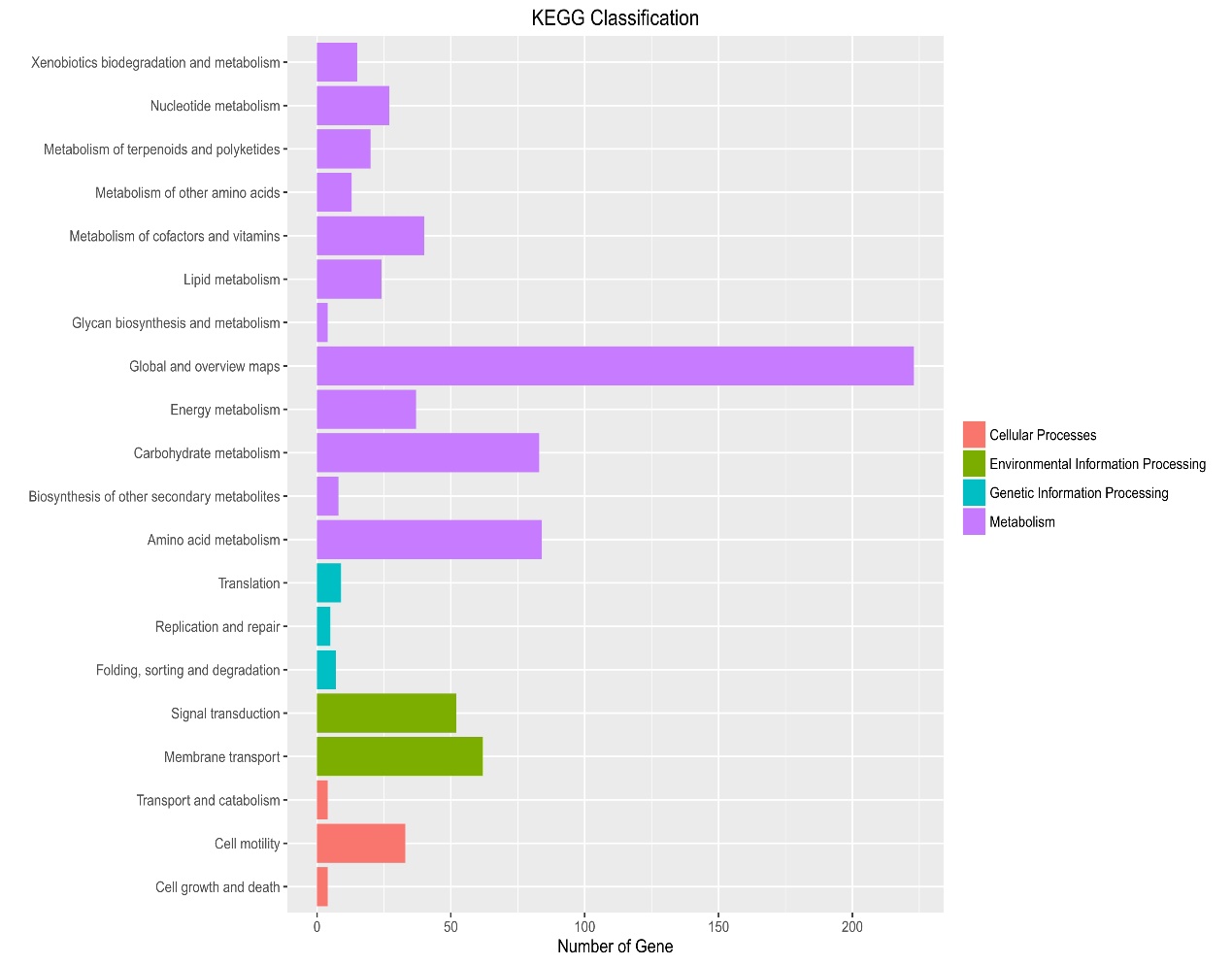


**Fig. S5 Analysis of DGE (difference of gene expression) between WH1 and Δ*srfA*. (A):** The horizontal and vertical coordinates represent the expression level (FPKM value) of the genes transcription in WH1 and Δ*srfA*, respectively. The value of the vertical and horizontal coordinates is Log2 logarithmic processing. Each point represents a specific gene transcription, and the red dots indicate the significant up-regulation of genes transcription, the blue dots indicate the significantly down-regulated genes transcription, and the gray points indicate the genes transcription without significant difference between WH1 and Δ*srfA*. **(B):** Top 30 of GO (gene ontology) enrichment between WH1 and Δ*srfA*. Rich Factor refers to the ratio of genes number located in the GO entry to the total number of genes. The larger Rich Factor means the greater degree of enrichment. Q-value is the *p*-value after multiple hypothesis verification, and the smaller value means the more significant enrichment. **(C):** KEGG pathway classification of DGE between WH1 and Δ*srfA*.

Analysis by transcriptomes, we found the DGE (Difference of gene expression) was very different between WH1 and Δ*srfA*. Most of DGE were up-regulated in Δ*srfA* compared to WH1 (A). GO (Gene ontology) was also used for analysis of DGE between WH1 and Δ*srfA*. There were total 3477 genes involved in 39 GOs with transcriptional difference between these two strains. Among them, 305 genes were involved in cellular component, 914 genes in molecular function, and 2258 genes in biological process. The genes involved in cellular process, metabolic process, single organism process, and catalytic activity were the richest among all genes with transcriptional difference. Further GO enrichment analysis by GOstats showed total 1199 genes enriched in 92 GOs between WH1 and Δ*srfA*. In the biological process, there were several processes including pyrimidine ribonucleotide biosynthesis, several amino acid biosynthesis (arginine, histidine, proline, etc.), amino acid catabolic process, cellular response to phosphate starvation, polysaccharide biosynthesis, amino acid transport, etc., with a Rich Factor more than 2.0. In the molecular function, there were several functions including amino acid-transporting ATPase, carbamoyl-phosphate synthase (glutamine-hydrolyzing), oligopeptide transporter, deacetylase, efflux transmembrane transporter, phosphate ion transmembrane-transporting ATPase, amino acid transmembrane transporter, etc., with a Rich Factor more than 2.0 (B).

On the basis of analysis of GO enrichment, we further analyzed the difference of genes transcription between WH1 and Δ*srfA* by KEGG (Kyoto Encyclopedia of Genes and Genomes). There were total 400 DGEs including metabolism (270 genes), environmental information processing (111 genes), cellular processes (41 genes) and genetic information processing (21 genes) between WH1 and Δ*srfA* (C). Among them there were 223 major DGEs involved in antibiotic biosynthesis, biosynthesis of secondary metabolites, carbon metabolism, energy metabolism, amino acid metabolism, etc. In detail, there were 84 DGEs for amino acid metabolism, 83 DGEs for carbohydrate metabolism such as EMP and TCA, 52 DGEs for signal transduction such as two-component system, and 62 DGEs for membrane transport such as PTS and ABC transporter.

**Figure S6**


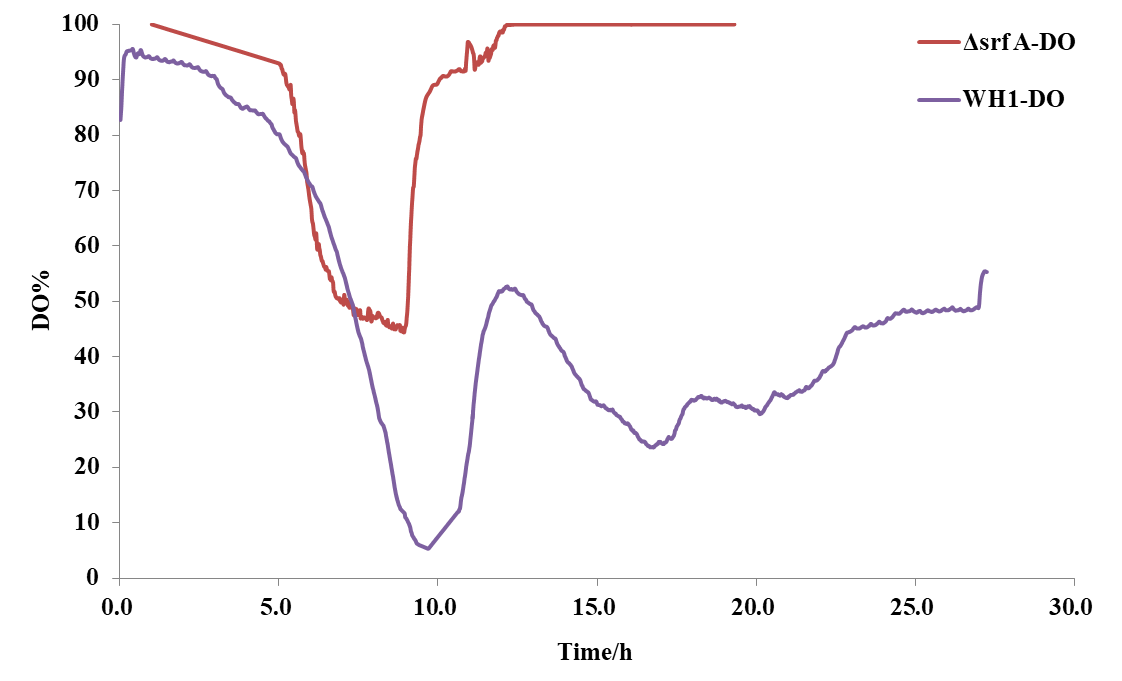


**Fig. S6 Dissolved O_2_ (DO) in culture (WH1 *vs*. Δ*srfA*).**

**Figure S7**

(A)

(B)

**Fig. S7 Comparison of the genes transcription involved in chemotaxis and motility between Δ*srfA* and WH1.** **(A):** Transcription of genes involved in chemotaxis; **(B):** Transcription of genes involved in motility. Double stars mean significant difference (*p*<0.01) between Δ*srfA* and WH1.

**Figure S8**

Δ*srfA*  WH1


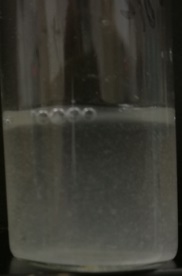

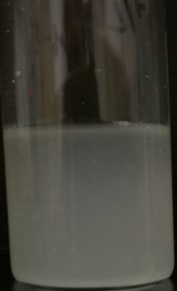


**Fig. S8. Δ*srfA* cultured in M9 medium.** The modified M9 medium contained mixed sugars (0.10 g/L glucose and 3.90 g/L xylose) was used for culturing WH1 and Δ*srfA*, respectively. After glucose exhaustion, WH1 could use xylose for growth while Δ*srfA* could not due to lack of surfactin.**Figure S9**

(A) (B)

M 1 2 M 1 2


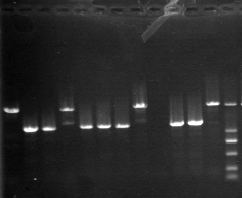

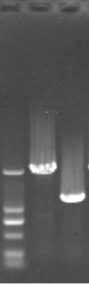


2000

1000

2000

1000

**Fig. S9 PCR verification of Δ*ccpA* and Δ*srfA*Δ*ccpA.*** **(A):** Δ*ccpA*. Lane 1 was the PCR product from WH1, and lane 2 was the PCR product from the strain with double crossover for deletion of *ccpA*. **(B):** Δ*srfA*Δ*ccpA*. Lane 1 was the PCR product from Δ*srfA*, and lane 2 was the PCR product from the strain with double crossover of *ccpA* in Δ*srfA*.
